# Image vaccine against steganography in encrypted domain

**DOI:** 10.1038/s41598-025-88384-8

**Published:** 2025-02-03

**Authors:** Xinran Li, Zichi Wang

**Affiliations:** 1 Business School, University of Shanghai for Science and Technology, Shanghai, 200093 China; 2 School of Communication and Information Engineering, Shanghai University, Shanghai, 200444 China

**Keywords:** Vaccine, Steganography, Encrypted images, Electrical and electronic engineering, Imaging and sensing

## Abstract

This paper investigates on the defense against steganography, and the overall purpose of the study is to design a satisfactory defense scheme in encrypted domain. Image vaccine against steganography is an effective technique to discover the utilization of steganography with extremely high detection accuracy. However, the image owner and vaccine provider are not the same person usually. To meet the requirements of steganography defense and privacy protection simultaneously, this paper proposes a vaccine scheme against steganography for encrypted images. After encrypting the entire data of a original image using a stream cipher, the vaccine data can be injected into the image without knowing the image content. With an encrypted image containing vaccine data, one can decrypt it to obtain the vaccinated image. When steganography is executed on vaccinated image, the utilization of steganography can be discovered in encrypted domain. Experimental results show that the detection accuracy of our scheme on steganography is 100% for all cases. That means the utilization of steganography can be always detected using our scheme. Integrate image vaccine into the imaging process of digital cameras in IoT systems is a potential practical application of our scheme. Non-universal detection mechanism is the potential limitations of this study, and it may be solved by pre-processing original image instead of injecting specific data.

## Introduction

Communication achieves the sharing of knowledge and expertise, enabling individuals to learn from each other and grow professionally. It has been well developed^1^. Steganography is a technique aims to achieve covert communication by embedding secret data into normal media. Secret data is embedded into a given cover media by slightly modifying the media content such as pixel values. The cover media is used to carry secret data, and so the media with enormous quantity can be used as the cover for steganography. In recent decades, the widespread digital images^2^ became a popular kind of cover media for steganography. The epidemic utilization of steganography is a huge threat to information security, since illicit content can be transmitted secretly. In this case, defensive strategies against steganography should be developed. As the adversarial counterpart, the technique of steganalysis is developed for identifying possible covert communication of steganography^3^. Given a suspicious image, statistical properties of image elements are calculated to distinguish clean and stego images.

Except steganalysis, recently published image vaccine is also an effective technique against steganography^4^. In the process of image vaccine, vaccine data is injected into an image for protection. Then, the obtained vaccinated image becomes immune to steganography. When secret data is embedded into vaccinated image, the existence of secret data can be always discovered by verifying the vaccine data. But it is designed for plaintext images. Image vaccination may be a private technique which is controlled by a vaccine provider. For profit reason, a vaccine provider is unwilling to publish the details of vaccine injection and verification. As a result, the image owner does not know how to inject vaccine data into his images, and then he has to ask the vaccine provider for vaccine injection and verification. Therefore, the image owner and vaccine provider are not the same person usually. Thus, image owner need to send the original image to vaccine provider for injecting vaccine data. For privacy protection, the image owner hopes that the vaccine provider can inject vaccine data into the original image without knowing the image content. Therefore, a vaccine scheme for encrypted image is desirable. However, to the best of our knowledge, an image vaccine scheme for encrypted image has not been reported in the literature.

**Figure 1:**
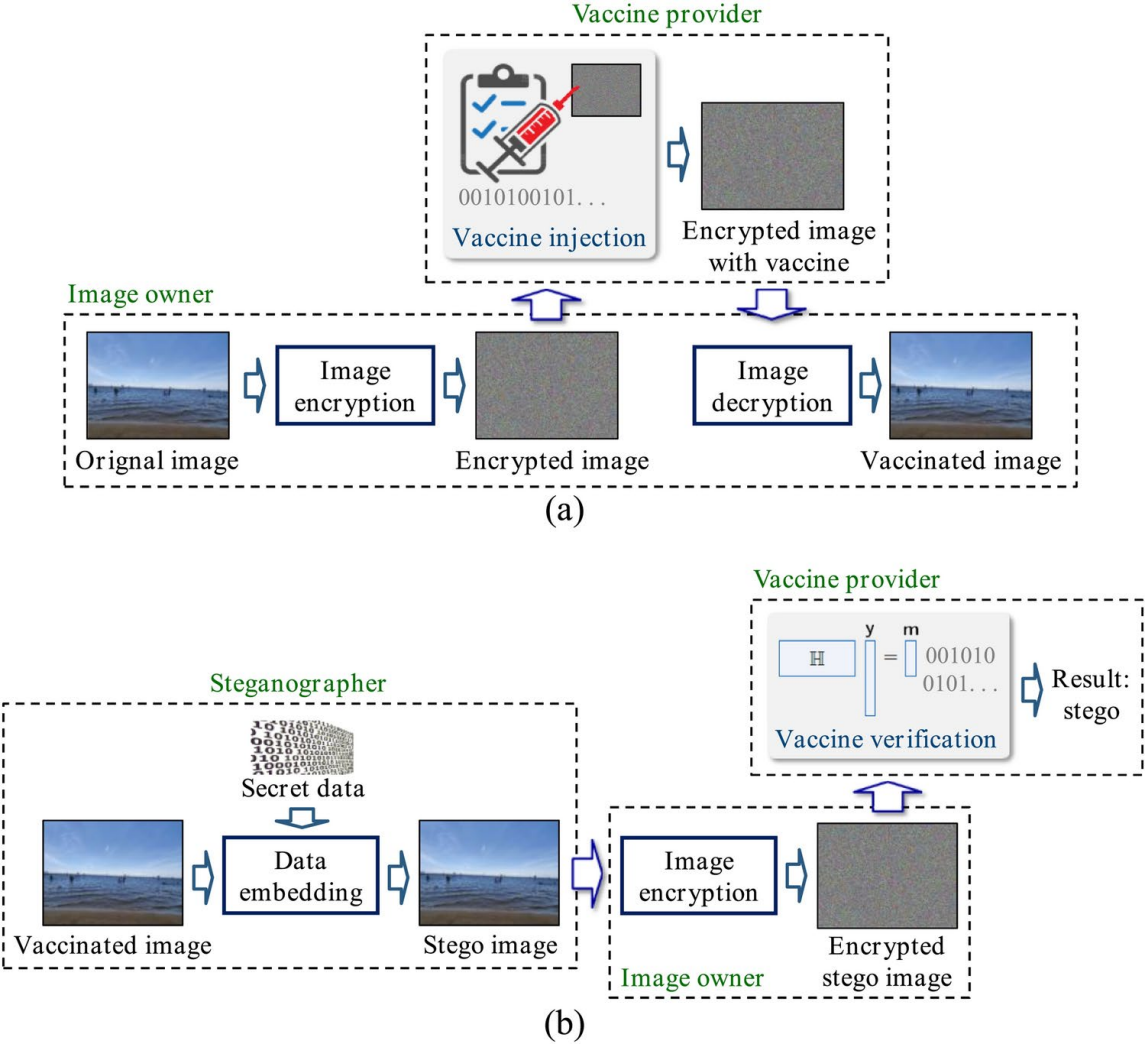
The idea of image vaccine in encrypted domain, (**a**) vaccine injection, (**b**) vaccine verification.

In this paper, we propose an image vaccine scheme against steganography in encrypted domain. As shown in Fig. 1a, an image owner encrypts the original image and then a vaccine provider injects vaccine data into the encrypted image though he does not know the original content. With an encrypted image containing vaccine data, the image owner can decrypted it and the obtained image is the vaccinated image. When secret data is embedded into vaccinated image, the existence of secret data can be discovered in encrypted domain. As shown in Fig. 1b, the image owner encrypts the image to be verified and then send it to the vaccine provider. The vaccine provider executes vaccine verification without knowing the image content. That means both the processes of vaccine injection and vaccine verification are executed in encrypted domain.

On the whole, existing methods for steganography defense can be classified into two categories: steganalysis and image vaccine. For steganalysis, it still has not achieved a high level especially for small payloads of steganography. While for existing image vaccine, it is not designed for encrypted image. The contributions of this paper are listed as follows:

1. We propose the first vaccine scheme against steganography for encrypted images. Using our scheme, both vaccine injection and vaccine verification are executed in encrypted domain, and so steganography defense and privacy protection can be achieved simultaneously.
2. The detection accuracy of our scheme on steganography is 100%, which is the same as the vaccine scheme against steganography for plaintext images. It means that our scheme adds the function of privacy protection without decreasing the performance of defending steganography.

The rest of this paper is organized as follows. We introduce the related work in section Related work. Our vaccine method for encrypted images is described in section Proposed method. Experimental results and analysis are provided in section Experimental results. Section Conclusion concludes the whole paper.

## Related work

In this section, we introduce some related work, including digital image steganography, image vaccine against steganography and image processing in encrypted domain.

### Digital image steganography

Digital image steganography aims to transmit data secretly via normal images. To achieve this, secret data is embedded into a cover image by slightly modifying image elements such as pixels for spatial images^5^ or DCT coefficients for JPEG images^6^. At the beginning, various steganographic codings are designed to decrease the number of modifications. At present, the most popular framework of digital image steganography is embedding distortion minimization^7–9^ with a user-defined distortion function. In the framework, performance of the part of steganographic coding is close to theoretical bound. For this reason, researchers focus on the design of distortion function, e.g., SUNIWARD (Spatial Universal Wavelet Relative Distortion)^10^, MiPOD (Minimizing the Power of Optimal Detector)^11^, HILL (High-pass, Low-pass, and Low-pass)^12^ for spatial images, and JUNIWARD (JPEG Universal Wavelet Relative Distortion)^10^, UERD (Uniform Embedding Revisited Distortion)^13^, GUED (Generalized UED)^14^ for JPEG images.

In our scheme, the utilization of above-mentioned steganographic methods can be discovered in encrypted domain. It contribute to prevent epidemic utilization of steganography and make contribution to information security.

### Image vaccine against steganography

Image vaccine against steganography is an effective technique to discover the utilization of steganography. For a given image to be protected, a small quantity of vaccine data is injected. The obtained vaccinated image will be immune to steganography, i.e, sensitive to the modification operations of steganography. When secret data is embedded into the vaccinated image, the existence of secret data can be discovered by verifying the vaccine data. In^4^, The fragility of distortion minimization coding is used to verify the injected vaccine data. When steganography is executed on the vaccinated image, the vaccine data cannot be correctly extracted due to the fragility of distortion minimization coding, and so the extraction error of vaccine data is the proof of the existence of secret data.

Usually, the image owner and vaccine provider are not the same person. For privacy protection, we propose to execute image vaccine in encrypted domain. In this way, both steganography defense and privacy protection can be achieved simultaneously.

### Image processing in encrypted domain

In order to protect the content privacy, digital images are transmitted and stored with encrypted version. However, during the transmission or the archiving of encrypted images, it is often necessary to process them without knowing the plaintext content^15^. For this reason, image processing in encrypted domain has been concerned in recent decades. Some researchers developed the field of reversible data hiding in encrypted domain^16–19^. A content owner encrypts original image and then upload it to the server. At the server side, some additional data is embedded into the encrypted image without decryption. At the receiver side, additional data can be correctly extracted and the original image can be perfectly recovered. To obtain image statistical properties securely, feature extraction in encrypted domain has been developed^20,21^. In^20^, the feature with co-occurrence matrix used for steganalysis is extracted from encrypted images by designing specific encryption algorithm. In^21^, the authors generated robust image hashing in encrypted domain using homomorphic cipher. In addition, a number of methods about secure outsourcing^22^, discrete wavelet transform in encrypted domain^23^, and facial expression recognition in encrypted domain^24^ have also been designed.

Currently, an image vaccine scheme in encrypted domain has not been reported in the literature. In this paper, we propose a vaccine scheme against steganography for encrypted images. Vaccine injection and verification can be achieved in encrypted domain.

## Proposed method

**Figure 2:**
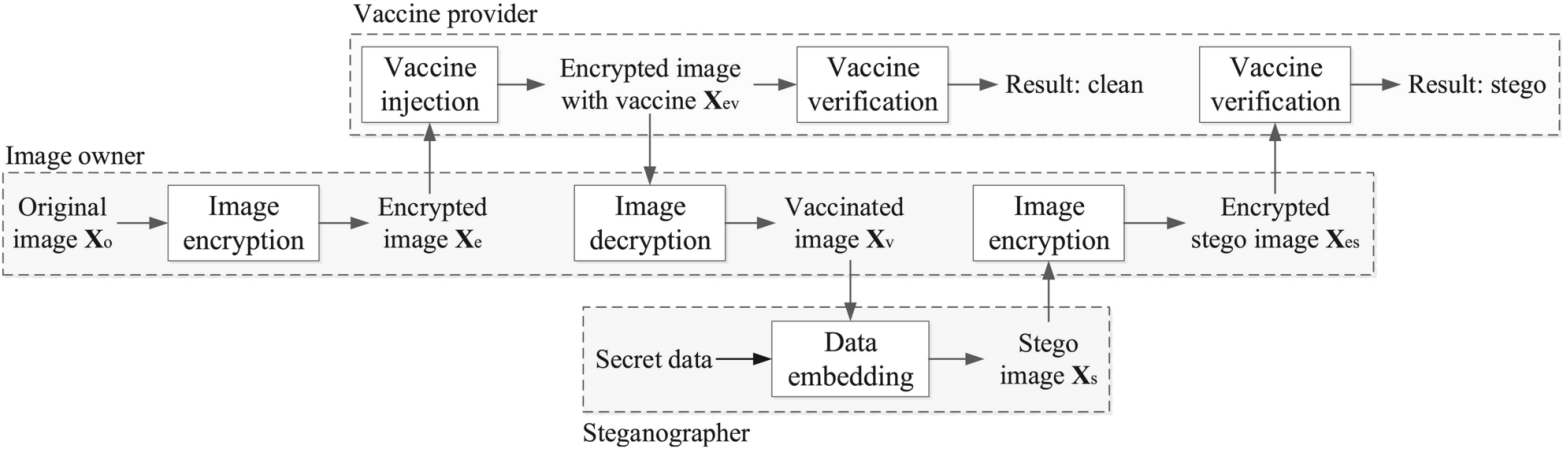
Architecture of proposed image vaccine in encrypted domain.

Image vaccine is effective in discovering the utilization of steganography. In this section, we propose a scheme to achieve image vaccine in encrypted domain. Both the processes of vaccine injection and vaccine verification are executed in encrypted domain. The architecture of our scheme is shown in Fig. 2. An image owner encrypts the original image $\textbf{X}_o$, and then sends the encrypted image $\textbf{X}_e$ to the vaccine provider. On the vaccine provider side, vaccine data can be injected into $\textbf{X}_e$ without knowing the original content. After vaccine injection, the vaccine provider returns the encrypted image containing vaccine data $\textbf{X}_{ev}$ to the image owner. The image owner directly decrypts $\textbf{X}_{ev}$ and the obtained image is the vaccinated image $\textbf{X}_v$. When steganography is executed on $\textbf{X}_v$, i.e., secret data is embedded into $\textbf{X}_v$, the existence of secret data in stego image $\textbf{X}_s$ can be discovered in encrypted domain. Specifically, the image owner encrypts $\textbf{X}_s$ and then sends image $\textbf{X}_{es}$ to the vaccine provider. On the vaccine provider side, vaccine verification can be executed without knowing the image content. Finally, the vaccine provider returns the decision to the image owner.

In practice, when the image owner saw his image elsewhere. He can verify the fact that whether his image has been used for steganography with the help of vaccine provider, as long as vaccine data is injected into the image before. He encrypts the image and then vaccine provider executes vaccine verification and returns the decision (“clean” or “stego”). If the image has not been used for steganography, the decision will be “clean”. Otherwise, the decision will be “stego”. Notations in this paper are listed in Table 1.

**Table 1:** Notation list in this paper.

Notations	Meanings	Notations	Meanings
$\textbf{X}_o$	Original image	$x_o(i)$	Pixel of original image
$\textbf{X}_e$	Encrypted image	$x_e(i)$	Pixel of encrypted image
$\textbf{X}_{ev}$	Encrypted image containing vaccine data	$x_{ev}(i)$	Pixel of encrypted image containing vaccine data
$\textbf{X}_v$	Vaccinated image	$x_v(i)$	Pixel of vaccinated image
$\textbf{X}_s$	Stego image	$x_s(i)$	Pixel of stego image
$\textbf{X}_{es}$	Encrypted stego image	$x_{es}(i)$	Pixel of encrypted stego image
$b^k_o(i)$	The *k*+1 bit of $x_o(i)$	$b^k_e(i)$	The *k*+1 bit of $x_e(i)$
$b^k_{ev}(i)$	The *k*+1 bit of $x_{ev}(i)$	$b^k_v(i)$	The *k*+1 bit of $x_v(i)$
$b^k_v(i)$	The *k*+1 bit of $x_v(i)$	$b^k_{es}(i)$	The *k*+1 bit of $x_{es}(i)$
$r^k(i)$	Pseudo-random bit	$\rho _e(i)$	Modification cost of pixel $x_e(i)$
*m*	Bits of vaccine data	$p_e(i)$	Probability to modify $x_e(i)$
$\textbf{L}_{ev}$	The LSBs of $\textbf{X}_{ev}$	$\textbf{H}$	Low-density parity-check matrix for vaccine injection

### Image encryption

Before vaccine injection, the image owner would like to encrypt the original image $\textbf{X}_o$. Assume $\textbf{X}_o$ composed of *n* pixels $\{x_o(1), x_o(2),\ldots , x_o(n)\}$, and each pixel with gray value falling into [0, 255] is represented by 8 bits. Denote the bits of pixel $x_o(i)$ as $b^k_o(i)$, $i\in \{1, 2,\ldots , n\}$, $k\in \{0, 1,\ldots , 7\}$. That means,

1 $$\begin{aligned} b^k_o(i)=\textrm{mod}\left[ \lfloor \frac{ {x_o(i)}}{2^{ {k}}}\rfloor , 2\right] , \end{aligned}$$

and

2 $$\begin{aligned} x_o(i)=\sum _{k=0}^7 b^k_o(i)\cdot 2^k, \end{aligned}$$

where “$\textrm{mod}[\cdot ]$” and “$\lfloor \cdot \rfloor$” represent the modulo and round down operations, respectively.

During image encryption, a stream cipher is employed to encrypt each bit $b^k_o(i)$. Stream cipher is a symmetric encryption technique where a pseudo-random sequence of bits is generated and combined with (such as exclusive-or operation) plaintext data to produce ciphertext. This process is reversible, meaning the same pseudo-random bits can be used to decrypt the ciphertext back into plaintext. The pseudo-random bits are generated by a keystream generator, typically based on a shorter initial key, which is expanded into a longer sequence through a complex algorithm. Stream ciphers can be categorized into synchronous and self-synchronizing types. Synchronous stream cipher generates a pseudorandom number stream independently of the plaintext and ciphertext messages. Self-synchronizing stream cipher updates its state based on previous ciphertext digits. The receiver automatically synchronizes with the keystream generator after receiving a certain number of ciphertext digits. Specifically, the encrypted bit $b^k_e(i)$ is the exclusive-or result of $b^k_o(i)$ and a pseudo-random bit $r^k(i)$, as shown in Eq. (3).

3 $$\begin{aligned} b^k_e(i)=b^k_o(i) \oplus r^k(i), \end{aligned}$$

where $r^k(i)$ is generated by an encryption key using a standard stream cipher. Then, all the encrypted bits are concatenated orderly to form the encrypted image $\textbf{X}_e$. Anyone without the encryption key cannot obtain the same $r^k(i)$, and so cannot decrypt $b^k_e(i)$ into $b^k_o(i)$. On the contrary, with the correct encryption key, one can obtain $b^k_o(i)$ from $b^k_e(i)$ by generating the same $r^k(i)$ and calculating the exclusive-or result of $B^k_o(i)$ and $r^k(i)$, as shown in Eq. (4).

4 $$\begin{aligned} b^k_o(i)=b^k_e(i) \oplus r^k(i), \end{aligned}$$

Then, the image owner sends $\textbf{X}_e$ to the vaccine provider for vaccine injection. Certainly, vaccine provider cannot obtain any information about $\textbf{X}_o$ from the encrypted image $\textbf{X}_e$.

### Vaccine injection

The process of vaccine injection is executed in encrypted domain. In our scheme, the distortion minimization framework^7^ as discussed in section Digital image steganography is employed. In the framework, a modification cost is assigned for each image pixel to quantify the image distortion caused by modifying the pixel. Since vaccine data is injected into the encrypted image $\textbf{X}_e=[x_e(1), x_e(2),\ldots , x_e(n)]^\textrm{T}$, denote the modification cost of pixel $x_e(i)$ as $\rho _e(i)$, then the theoretical minimal distortion $D(\textbf{X}_e,\textbf{X}_{ev})$ between encrypted image $\textbf{X}_e$ and the encrypted image containing vaccine data $\textbf{X}_{ev}$ with *m* bits of vaccine data can be calculated as Eq. (5):

5 $$\begin{aligned} D(\textbf{X}_e,\textbf{X}_{ev})=\sum _{i=1}^{k} p_e(i)\rho _e(i), \end{aligned}$$

where

6 $$\begin{aligned} p_e(i)=\frac{e^{-\lambda \rho _e(i)}}{1+e^{-\lambda \rho _e(i)}}, \end{aligned}$$

is the probability to modify $x_e(i)$, and $\lambda > 0$ is used to let the information entropy of modifying probabilities equal to *m*, as shown in Eq. (7).

7 $$\begin{aligned} -\sum _{i=1}^{n} \{p_e(i)log_2p_e(i)+[1-p_e(i)]log_2[1-p_e(i)]\}=m. \end{aligned}$$

To approximate the theoretical bound $D(\textbf{X}_e,\textbf{X}_{ev})$, some distortion minimization codings have been designed, i.e., STC^7^, SPC^8^, and log-BPGD^9^. In our scheme, the popular STC coding is employed. Using STC, vaccine data $\textbf{v}=[v(1), v(2), \ldots , v(m)]^\textrm{T}\in \{0,1\}^m$ can be injected into $\textbf{X}_e$ by modifying the pixels to meet Eq. (8).

8 $$\begin{aligned} \textbf{H}\textbf{L}_{ev}=\textbf{v}, \end{aligned}$$

9 $$\begin{aligned} l_{ev}(i)=mod[x_{ev}(i), 2]. \end{aligned}$$

where $\textbf{L}_{ev}=[l_{ev}(1), l_{ev}(2),\ldots , l_{ev}(n)]^\textrm{T}$ is the LSBs (least significant bits) of $\textbf{X}_{ev}$, $l_{ev}(i)\in \{0, 1\}$. Matrix $\textbf{H}\in \{0, 1\}^{m\times n}$ is a low-density parity-check matrix that is determined by the speed and efficiency of vaccine injection. All operations are conducted with binary arithmetic. Thus, vaccine data $\textbf{v}$ can be directly extracted through the matrix computation in Eq. (8). Since there are a number of solutions for Eq. (8), the one corresponding to the minimum distortion is used as the optimal solution.

Thus, vaccine data $\textbf{v}$ can be injected into $\textbf{X}_e$ with modification costs $\{\rho _e(1), \rho _e(2),\ldots , \rho _e(n)\}$. There have a number of algorithms to determine the cost values for plaintext image as described in section Digital image steganography. Usually, costs of smooth areas are greater than those of complex areas. Since a greater cost results in smaller modification probability as shown in Eq. (6), the modifications on smooth areas will be lesser than those on complex areas. In this way, the modification trace is hard to be detected. In our scheme, the image used for vaccine injection is encrypted. The information about image content is unknown. For this reason, it cannot find a criterion to assign different costs for different pixels. Therefore, the cost values are set as constant in our scheme. That means $\rho _e(1)=\rho _e(2)=\ldots =\rho _e(n)=1$.

### Image decryption

With the encrypted image containing vaccine data $\textbf{X}_{ev}$, the image owner can directly decrypts $\textbf{X}_{ev}$ to obtain the vaccinated image $\textbf{X}_v$. Denote the *n* pixels of $\textbf{X}_{ev}$ and $\textbf{X}_v$ as $\{x_{ev}(1), x_{ev}(2),\ldots , x_{ev}(n)\}$ and $\{x_v(1), x_v(2),\ldots , x_v(n)\}$, then the bits $b^k_{ev}(i)$ and $b^k_v(i)$ of pixels $x_{ev}(i)$ and $x_v(i)$ are,

10 $$\begin{aligned} b^k_{ev}(i)=\textrm{mod}\left[ \lfloor \frac{ {x_{ev}(i)}}{2^{ {k}}}\rfloor , 2\right] , \end{aligned}$$

and

11 $$\begin{aligned} b^k_v(i)=\textrm{mod}\left[ \lfloor \frac{ {x_v(i)}}{2^{ {k}}}\rfloor , 2\right] , \end{aligned}$$

To obtain $x_v(i)$, the image owner generates the pseudo-random bit $r^k(i)$ using encryption key, and then calculates the exclusive-or result of $b^k_{ev}(i)$ and $r^k(i)$, as shown in Eq. (12).

12 $$\begin{aligned} b^k_v(i)=b^k_{ev}(i) \oplus r^k(i), \end{aligned}$$

Then, all the decrypted bits are concatenated orderly to form the vaccinated image $\textbf{X}_v$. Image $\textbf{X}_v$ is different with the original image $\textbf{X}_o$ since a part of pixels are modified during vaccine injection. Although vaccine injection is executed in encrypted domain, the modification trace has been reserved in the plaintext domain due to the exclusive-or operation. The quality of vaccinated image will be discussed in section Image quality.

### Vaccine verification

With the vaccinated image $\textbf{X}_v$, the utilization of steganography can be discovered by vaccine verification. When steganography is executed on $\textbf{X}_v$, i.e., secret data is embedded into $\textbf{X}_v$, the existence of secret data in stego image $\textbf{X}_s$ can be discovered. The process of vaccine verification is also executed in encrypted domain, since vaccine data is injected in encrypted domain. Denote the image to be verified as $\textbf{X}_t$, the image owner encrypts $\textbf{X}_t$ and then sends the obtained image $\textbf{X}_{et}$ to the vaccine provider for vaccine verification. Image $\textbf{X}_t$ should be same as $\textbf{X}_v$ if steganography has not executed, or should be same as $\textbf{X}_s$. In encrypted domain, image $\textbf{X}_{et}$ should be same as $\textbf{X}_{ev}$ if steganography has not executed, or should be same as $\textbf{X}_{es}$.

During vaccine verification, the vaccine provider attempts to extract vaccine data from the encrypted image $\textbf{X}_{et}$. Specifically, the matrix computation in Eq. (8) is executed on $\textbf{X}_{et}$. That is,

13 $$\begin{aligned} \textbf{H}\textbf{L}_{et}=\textbf{u}, \end{aligned}$$

where $\textbf{u}$ is the extracted data obtained from $\textbf{X}_{et}$, and $\textbf{L}_{et}=[l_{et}(1), l_{et}(2),\ldots , l_{et}(n)]^\textrm{T}$ is the LSBs of $\textbf{X}_{et}$, $l_{et}(i)=mod[x_{et}(i), 2]$. Then $\textbf{u}$ is compared with the injected vaccine data $\textbf{v}$. If the image $\textbf{X}_t$ has not been used for steganography, $\textbf{X}_t$ has not been modified, and so its encrypted version $\textbf{X}_{et}$ will be same as $\textbf{X}_{ev}$. Thus, $\textbf{u}$ will be same as $\textbf{v}$, and it means $\textbf{X}_t$ is “clean”. Otherwise, when steganographic operation is executed on $\textbf{X}_t$ (that means $\textbf{X}_t$ is $\textbf{X}_s$), a part of pixels in $\textbf{X}_t$ will be modified including a part of LSBs. In this case, the encrypted version $\textbf{X}_{et}$ of $\textbf{X}_t$ will be different from that of $\textbf{X}_v$ due to the exclusive-or operation of encryption. Thus, the result $\textbf{u}$ of Eq. (13) will be different from the result $\textbf{v}$ of Eq. (8), and it means $\textbf{X}_t$ is “stego”.

### Examples of main processes

To help readers better understand the implementation, this subsection gives some examples of the embedding and extraction processes, as well as the encryption and decryption processes. Examples are given according to the order of the procedures in Fig. 2. With a original image $\textbf{X}_o$, as shown in Eq. (14) for example, an image owner encrypts $\textbf{X}_o$ using Eq. (3). Then, $\textbf{X}_e$ is obtained, as shown in Eq. (15). It can be seen that encrypted image is essentially different from the original version. None of the information of original images can be captured from the encrypted image, since the encrypted values involve the whole range.

14 $$\begin{aligned} \textbf{X}_o= \begin{bmatrix} 189 & 192 & 190 & 190 \\ 190 & 194 & 191 & 189 \\ 191 & 190 & 188 & 187 \\ 187 & 193 & 186 & 191 \end{bmatrix}, \end{aligned}$$

15 $$\begin{aligned} \textbf{X}_e= \begin{bmatrix} 124 & 222 & 171 & 240 \\ 177 & 160 & 196 & 107 \\ 24 & 0 & 201 & 59 \\ 186 & 170 & 60 & 206 \end{bmatrix}. \end{aligned}$$

An image owner sends $\textbf{X}_e$ to the vaccine provider. On the vaccine provider side, vaccine data with 10 bits is injected into $\textbf{X}_e$ without knowing the original content using Eq. (8). After vaccine injection, the vaccine provider returns the encrypted image containing vaccine data $\textbf{X}_{ev}$ to the image owner, as shown in Eq. (16).

16 $$\begin{aligned} \textbf{X}_{ev}= \begin{bmatrix} 124 & 222 & 171 & 239 \\ 177 & 160 & 197 & 108 \\ 23 & 1 & 202 & 59 \\ 187 & 169 & 61 & 206 \end{bmatrix}, \end{aligned}$$

It is clear that $\textbf{X}_{ev}$ is similar to $\textbf{X}_e$. That means the operation of vaccine injection has minor effect on the given image. Then, the image owner directly decrypts $\textbf{X}_{ev}$ using Eq. (12) and the obtained image is the vaccinated image $\textbf{X}_v$, as shown in Eq. (17).

17 $$\begin{aligned} \textbf{X}_v= \begin{bmatrix} 189 & 192 & 190 & 161 \\ 190 & 194 & 190 & 186 \\ 176 & 191 & 191 & 187 \\ 186 & 194 & 187 & 191 \end{bmatrix}. \end{aligned}$$

When steganography is executed on $\textbf{X}_v$, i.e., secret data is embedded into $\textbf{X}_v$, stego image $\textbf{X}_s$ is obtained, as shown in Eq. (18).

18 $$\begin{aligned} \textbf{X}_s= \begin{bmatrix} 189 & 192 & 190 & 160 \\ 190 & 194 & 190 & 186 \\ 176 & 191 & 191 & 187 \\ 186 & 194 & 187 & 191 \end{bmatrix}, \end{aligned}$$

Using our scheme, the existence of secret data in $\textbf{X}_s$ can be discovered in encrypted domain. Specifically, the image owner encrypts $\textbf{X}_s$ using Eq. (3) and then sends image $\textbf{X}_{es}$ to the vaccine provider, as shown in Eq. (19).

19 $$\begin{aligned} \textbf{X}_{es}= \begin{bmatrix} 124 & 222 & 171 & 238 \\ 177 & 160 & 197 & 108 \\ 23 & 1 & 202 & 59 \\ 187 & 169 & 61 & 206 \end{bmatrix}. \end{aligned}$$

On the vaccine provider side, vaccine verification can be executed on $\textbf{X}_{es}$ without knowing the image content. In $\textbf{X}_{es}$, the existence of vaccine data cannot be detected. Thus, it can be concluded that image $\textbf{X}_s$ contains secret data. Comparing $\textbf{X}_v$ and $\textbf{X}_s$, we can see that only one pixel has been modified. In spite of this, our scheme is also effective.

## Experimental results

To verify the effectiveness of our method, a series of experiments are conducted in this section.

### Experiment setup

In experiments, we employed the image dataset UCID^25^ which contains 1338 uncompressed color images sized 512$\times$384. All the images in UCID were respectively used as the original image $\textbf{X}_o$ for encryption. After encryption, the obtained 1338 encrypted images ($\textbf{X}_e$) were used for vaccine injection. Quantity of vaccine data was set as 100, 200, 300, 400, and 500 bits, respectively. The operation of vaccine injection should not affect original image obviously. For this reason, hundreds of vaccine data are enough. Then, 1338$\times$5 = 6690 encrypted images containing vaccine data ($\textbf{X}_{ev}$) can be obtained. After image decryption, the obtained 6690 images were the vaccinated images ($\textbf{X}_v$), as presented in Table 2.

**Table 2:** Number of each kind of images.

Kinds of images	Quantity
$\textbf{X}_o$: Original image	1338
$\textbf{X}_e$: Encrypted image	1338
$\textbf{X}_{ev}$: Encrypted image containing vaccine data	6690 = 1338$\times$5
$\textbf{X}_v$: Vaccinated image	6690
$\textbf{X}_s$: Stego image	133,800 = 6690$\times$4$\times$5
$\textbf{X}_{es}$: Encrypted stego image	133,800

To verify the effectiveness of injected vaccine data, the popular steganographic methods SUNIWARD, HILL, MiPOD, and DFEI^26^ were employed to embed secret data into the vaccinated images ($\textbf{X}_v$) with payload 0.1, 0.2, 0.3, 0.4, and 0.5 bpp (bits per pixel), respectively. Thus, 6690$\times$4$\times$5 = 133800 stego images ($\textbf{X}_s$) can be obtained. Finally, the stego images were encrypted to obtain 133800 encrypted stego images ($\textbf{X}_{es}$). The four embedding strategies are based on the popular framework of embedding distortion minimization. In the framework, performance of the part of steganographic coding is close to the theoretical bound in Eq. (5). For this reason, the four embedding strategies focus on the design of distortion function. In other words, the four embedding strategies are designed to calculate a cost value for each image pixel. After that, data embedding is executed using STC coding with the calculated cost values. As discussed in section Digital image steganography, the utilization of these embedding strategies can be discovered in encrypted domain using our scheme.

**Figure 3:**
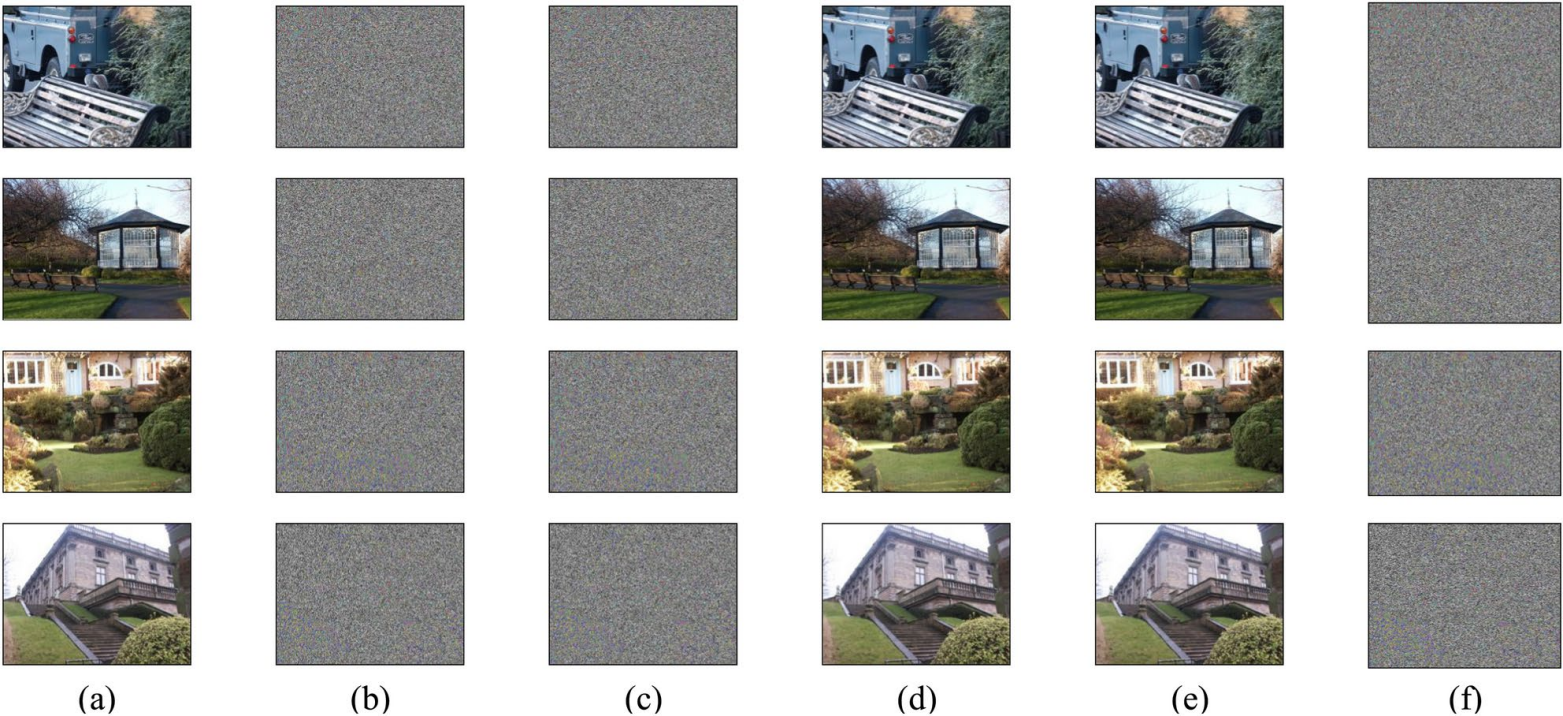
Images produced by our scheme, (**a**) original images, (**b**) encrypted images, (**c**) encrypted images containing vaccine data with 500 bits, (**d**) vaccinated images, (**e**) stego images using HILL with payload 0.1 bpp, (**f**) encrypted stego images. Images in (**a**), (**d**), (**e**) were obtained from the image dataset UCID, which is a public dataset at https://www.spiedigitallibrary.org/conference-proceedings-of-spie/5307/0000/UCID-an-uncompressed-color-image-database/10.1117/12.525375.short.

**Figure 4:**
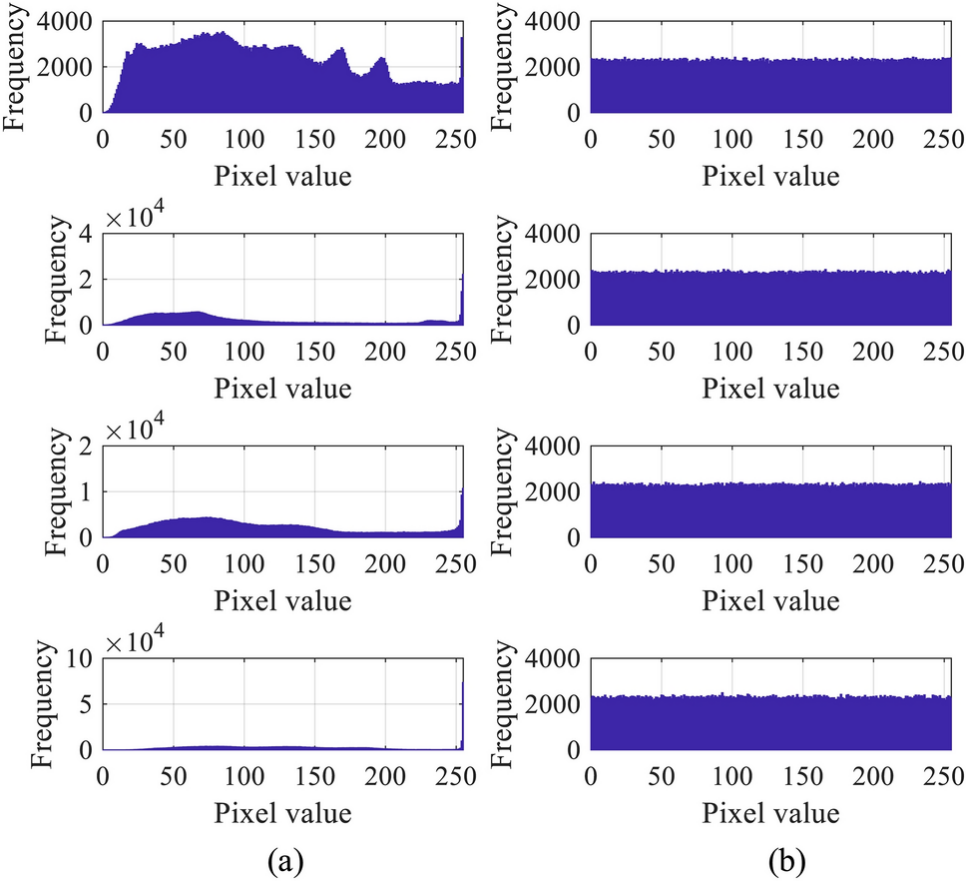
Histograms obtained from (**a**) original images, and (**b**) encrypted images.

### Image quality

Our vaccine scheme is designed for encrypted images. Both vaccine injection and vaccine verification are executed in encrypted domain. The security of image encryption should be guaranteed to for privacy protection. After image decryption, the injected vaccine data should not affect the quality of original image. To verify this, several images were respectively used as original image $\textbf{X}_o$, as shown in Fig. 3a. The encrypted versions of original images $\textbf{X}_e$ were shown in Fig. 3b. After vaccine data was injected with 500 bits, the corresponding encrypted image containing vaccine data $\textbf{X}_{ev}$ were shown in Fig. 3c, and the decrypted versions ($\textbf{X}_v$) were shown in Fig. 3d. Images in Fig. 3e were the corresponding stego images $\textbf{X}_s$ obtained using HILL with payload 0.1 bpp. Finally, Fig. 3f shows the encrypted stego images $\textbf{X}_{es}$.

It can be seen from Fig. 3b that the performance of image encryption is satisfactory since none of the information of original images can be captured from the encrypted image. The corresponding pixels histograms of the images in Fig. 3a and Fig. 3b were shown in Fig. 4. The results indicate that the histogram of the encrypted image were uniformly distributed in comparison with the original image. That means the Shannon entropy of the pixels of encrypted images were close to the maximal value. Thus, it is impossible to exploit them to obtain the content of original image. Therefore, the security of image encryption can be guaranteed.

**Table 3:** PSNR and SSIM values of vaccinated images.

	Images	Number of vaccine data (bits)				
		100	200	300	400	500
PSNR PSNR	Image 1	76.0	62.1	77.5	74.1	58.0
(dB)	Image 2	76.8	72.6	66.7	67.5	59.5
	Image 3	77.4	76.9	58.1	58.7	62.4
	Image 4	73.0	78.5	63.7	66.8	72.8
	UCID	77.5	72.3	69.2	67.7	65.9
SSIM	Image 1	1.0	0.9996	1.0	0.9998	0.9996
	Image 2	1.0	1.0	1.0	0.9999	1.0
	Image 3	1.0	1.0	1.0	1.0	0.9999
	Image 4	1.0	0.9999	0.9997	0.9999	0.9999
	UCID	0.9999	0.9999	0.9998	0.9998	0.9998

To examine the quality of vaccinated images, the indicators PSNR (Peak Signal-to-Noise Ratio) and SSIM (Structural Similarity Index Measurement)^27^ were employed. A higher value of PSNR or SSIM means better image quality, $0 < PSNR \leqslant +\infty$, $0 \leqslant SSIM \leqslant 1$. The PSNR and SSIM values between original and vaccinated images ($\textbf{X}_o$ and $\textbf{X}_v$) were listed in Table 3, where “Image 1”, “Image 2”, “Image 3”, “Image 4” respectively stand for the results of the four images in Fig. 3, and “UCID” stands for the average PSNR and SSIM values derived from the 1338 image pairs associated with UCID.

It is clear in Table 3 that the image quality is excellent since SSIM values were close to 1.0 which is the theoretical bound, and PSNR values were also large enough which corresponding to tiny modifications.

### Detection accuracy

The goal of image vaccine is to discover the utilization of steganography. Detection accuracy on steganography is the most important indicator for an image vaccine scheme. To verify detection accuracy of our scheme, all the 1338 images in UCID were used as the original images for encryption and vaccine injection. Then, the steganographic algorithms SUNIWARD, HILL, MiPOD, and DFEI were employed to embed secret data into the vaccinated images, respectively. Payload of steganography is set as 0.1, 0.2, 0.3, 0.4, and 0.5 bpp. Finally, the utilization of steganography was detected in encrypted domain by our scheme, detection accuracies (ratio of correctly decided) were listed in Table 4. Each detection accuracy was calculated among the corresponding encrypted images containing vaccine data ($\textbf{X}_{ev}$) and encrypted stego images ($\textbf{X}_{es}$).

As expected, the detection accuracy on steganography of our scheme is 100% for all cases, as shown in Table 4. That means for a given image, the decision on the utilization of steganography can be always correctly made by our scheme without knowing the content of original image. In other words, images $\textbf{X}_{ev}$ and $\textbf{X}_{es}$ can be always correctly classified by our method. The result is reasonable since the extraction error of vaccine data is not equal to zero even if only one pixel is modified. The vaccine data cannot be correctly extracted as long as $\textbf{X}_{ev}$ has been modified. On the other hand, modifications are necessary to the data embedding process of steganography even for small payload. The modifications in plaintext domain can be detected in encrypted domain as discussed in section Vaccine verification (the process of vaccine verification is executed in encrypted domain). Therefore, our scheme is effective to detect the utilization of steganography by capturing the trace of modification in encrypted domain. We achieve a perfect detection (100% accuracy) on steganography without knowing image content.

**Table 4:** Detection accuracy on steganography in encrypted domain (%).

Steganographic algorithms	Payload (bpp)	Number of vaccine data (bits)				
		100	200	300	400	500
DFEI	0.5	100.0	100.0	100.0	100.0	100.0
	0.4	100.0	100.0	100.0	100.0	100.0
	0.3	100.0	100.0	100.0	100.0	100.0
	0.2	100.0	100.0	100.0	100.0	100.0
	0.1	100.0	100.0	100.0	100.0	100.0
MiPOD	0.5	100.0	100.0	100.0	100.0	100.0
	0.4	100.0	100.0	100.0	100.0	100.0
	0.3	100.0	100.0	100.0	100.0	100.0
	0.2	100.0	100.0	100.0	100.0	100.0
	0.1	100.0	100.0	100.0	100.0	100.0
HILL	0.5	100.0	100.0	100.0	100.0	100.0
	0.4	100.0	100.0	100.0	100.0	100.0
	0.3	100.0	100.0	100.0	100.0	100.0
	0.2	100.0	100.0	100.0	100.0	100.0
	0.1	100.0	100.0	100.0	100.0	100.0
SUNIWARD	0.5	100.0	100.0	100.0	100.0	100.0
	0.4	100.0	100.0	100.0	100.0	100.0
	0.3	100.0	100.0	100.0	100.0	100.0
	0.2	100.0	100.0	100.0	100.0	100.0
	0.1	100.0	100.0	100.0	100.0	100.0

As described in section Related work, there are three related fields of our scheme, i.e., digital image steganography, image vaccine against steganography and image processing in encrypted domain. Our scheme is an adversarial technique of digital image steganography, and the corresponding results have been discussed above. Our scheme contribute to prevent epidemic utilization of steganography, which is also achieved in the image vaccine method against steganography in^4^. The detection accuracy on steganography of^4^ is also 100% for all cases, which is as well as that of our scheme. It is worth noting that the method in^4^ is designed for plaintext images. That means it cannot execute steganography dectection in encrypted images. Our proposal is an image vaccine scheme against steganography in encrypted domain, since the image owner and vaccine provider are not the same person usually. To the best of our knowledge, an image vaccine scheme in encrypted domain has not been reported in the literature.

Although a steganographer has a smartphone with a camera and can get an original innocent image, there are still a part of stego images obtained by embedding secret data into the images from others such as social networks. Since the non-detectable trace of vaccinated images, a steganographer does not know the image he obtained has been vaccinated. Ulteriorly, image vaccine can be integrated into the imaging process of digital cameras, as reported in^4^. When most of the digital cameras are equipped with image vaccine, the transmission of illegal content can be eradicated to a great extent. That means the images obtained by a smartphone with a camera are all vaccinated. Any steganographic operations executed on the vaccinated image will be discovered. In this case, images obtained from a camera and someone else have no essential difference. In order to realize that an image has been executed steganography, it is a feasible way to compare the transformed image and the original image. But in some cases, it is impractical to compare the transformed image and the original image, since the the original image may be inaccessible. Specifically, original images may be lost due to failures in storage media, corruption of files, or accidental deletion. For this reason, a special method steganography defense is useful, just in case.

## Conclusion

This paper proposes a vaccine scheme in encrypted domain to meet the requirements of steganography defense and privacy protection. Experimental results show that the vaccine detection accuracy is 100% which is the same as the vaccine scheme in plaintext domain. In our scheme, both vaccine injection and vaccine verification are executed in encrypted domain. Thus, the privacy of image content can be guaranteed. Therefore, the proposed scheme achieves the function of privacy protection without decreasing the performance of defending steganography. At the present stage, our scheme is not able to distinguish a steganographic transformation from an arbitrary image processing operation. It is a limitations of this study. In future work, some semi-fragile techniques can be employed to fill this deficiency.

## Data Availability

The datasets generated and/or analysed during the current study are constructed in the paper^25^, [https://www.spiedigitallibrary.org/conference-proceedings-of-spie/5307/0000/UCID-an-uncompressed-color-image-database/10.1117/12.525375.short].
